# Nutrient Acquisition, Rather Than Stress Response Over Diel Cycles, Drives Microbial Transcription in a Hyper-Arid Namib Desert Soil

**DOI:** 10.3389/fmicb.2019.01054

**Published:** 2019-05-14

**Authors:** Carlos León-Sobrino, Jean-Baptiste Ramond, Gillian Maggs-Kölling, Don A. Cowan

**Affiliations:** ^1^Centre for Microbial Ecology and Genomics, University of Pretoria, Pretoria, South Africa; ^2^Gobabeb Research and Training Centre, Gobabeb, Namibia

**Keywords:** desert actinobacteria, metatranscriptome analysis, chemoautotrophic CO_2_ fixation, diel activity cycles, stress resistance and tolerance, deserts and dryland ecosystems, soil microbial activity, RNA-seq

## Abstract

Hot desert surface soils are characterized by extremely low water activities for large parts of any annual cycle. It is widely assumed that microbial processes in such soils are very limited. Here we present the first metatranscriptomic survey of microbial community function in a low water activity hyperarid desert soil. Sequencing of total mRNA revealed a diverse and active community, dominated by Actinobacteria. Metatranscriptomic analysis of samples taken at different times over 3 days indicated that functional diel variations were limited at the whole community level, and mostly affected the eukaryotic subpopulation which was induced during the cooler night hours. High levels of transcription of chemoautotrophic carbon fixation genes contrasted with limited expression of photosynthetic genes, indicating that chemoautotrophy is an important alternative to photosynthesis for carbon cycling in desiccated desert soils. Analysis of the transcriptional levels of key N-cycling genes provided strong evidence that soil nitrate was the dominant nitrogen input source. Transcriptional network analyses and taxon-resolved functional profiling suggested that nutrient acquisition processes, and not diurnal environmental variation, were the main drivers of community activity in hyperarid Namib Desert soil. While we also observed significant levels of expression of common stress response genes, these genes were not dominant hubs in the co-occurrence network.

## Introduction

Arid lands (deserts) are defined as having a level of precipitation (P) below the potential evapotranspiration (PET) level (P/PET < 1). Such lands cover an estimated one-third of Earth’s terrestrial surface (Laity, 2008) and are projected to expand in current climate change scenarios (Reich et al., 2001). The Namib Desert, located along the western coast of Namibia and extending into southern Angola and northern South Africa, is the oldest (ca. 5 million years) continuously hyperarid (P/PET < 0.05) desert on Earth (Seely and Pallet, 2008).

According to current models, aridity results in habitat fragmentation, both geographically, leading to ”islands” of microbial biomass and diversity and, temporally, producing long periods of functional inactivity (Pointing and Belnap, 2012; Collins et al., 2014). Recent evidence from ATP measurements and rRNA transcript analyses suggest that some microorganisms remain active under these extreme conditions, although the active functions have not yet been detailed (Gunnigle et al., 2017; Schulze-Makuch et al., 2018).

In recent years, the microbial ecology of various Namib Desert edaphic niches has been extensively studied, highlighting the importance of water availability and soil chemistry (particularly phosphorus and ammonia) in microbial community assembly and function (Frossard et al., 2015; Ronca et al., 2015; Johnson et al., 2017; Scola et al., 2017).

RNA sequencing has been employed to study microbial community functional patterns in many different aquatic and terrestrial ecosystems. The short life-span and high turnover of messenger RNA (Belasco and Brawerman, 1993) allows ephemeral states of microbial communities to be captured without significant interference from legacy biomolecules or inactive microbial populations, as might be the case in 16S rRNA transcript-, DNA- or protein-based studies (Nielsen et al., 2006; Blazewicz et al., 2013). mRNA provides a better insight into the growth stage of prokaryotic communities than 16S RNA gene transcripts, as the later can also originate from dormant and/or recently deceased cells (Blazewicz et al., 2013). In desert environments, active prokaryotic communities have been identified by targeted 16S rRNA amplicon transcriptomics studies, demonstrating a diel pattern for several actinobacterial and proteobacterial taxa during dry periods (Gunnigle et al., 2017), and a dramatic activation of gamma-Proteobacteria, Firmicutes, and Bacteroidetes after rainfall (Štovíček et al., 2017).

In this study, we analyzed 12 shotgun metatranscriptomes from hyperarid desert soils sampled over 3 days. The experiment was designed to assess the diel transcriptional activity of desert edaphic microbial communities, particularly focusing on nutrient acquisition and stress response mechanisms. We also aimed to identify the key community members responsible for nutrient (C, N, P) cycling. Given the variations in light, temperature and humidity to which desert soil communities are exposed within a daily cycle, we hypothesized that functional transcriptional profiles would also show distinct diurnal cycles.

## Materials and Methods

### Sampling Procedure

The sampling site was located in the calcrete gravel plains of the central Namib Desert (23°33″34″S 15°02′25″E) (Scholz, 1972), Namibia, approximately 56 km from the coast. The mean annual precipitation at the site is estimated at 25 mm, principally derived from nocturnal marine fog (Eckardt et al., 2013). We implemented a 3 day sampling strategy with soil collection at near sunrise (6:00 h), at midday (12:00 h), at near sunset (18:00 h), and at midnight (24:00 h). A 10 × 10 m experimental plot was sub-divided into 64 quadrats (Supplementary Figure S1). Surface soils (0–4 cm) were collected at six hourly intervals at near sunrise, midday, near sunset and midnight (6:00, 12:00, 18:00, and 24:00 h) over 3 days from the 12th to the 14th April 2016, after a prolonged dry period (>1 year, Supplementary Table S1). Two Hygrochron iButton sensors (Embedded Data Systems, Lawrenceburg, KY, United States) were positioned at the corners of the plot, at ∼2 cm depth, recording temperature and relative humidity at 4 min intervals for the length of the experiment (Supplementary Figure S1). Soil respiration measurements were performed at the designated sampling times at four points within the plot using a LI-8100 IRGA (LI-COR Biosciences, Lincoln, NE, United States), covering an area of 83.7 cm^2^ with a 3 L chamber for 30 s (Supplementary Figure S1). Photosynthetically active radiation (PAR) was measured using a photometric sensor (Quantum, LI-COR) at the same internal plot locations. Surface soil samples (0–4 cm) were collected at six hourly intervals (6:00, 12:00, 18:00, and 24:00 h) over 3 days from the 12th to the 14th April 2016. Three randomly selected quadrats were sampled at each time point (Supplementary Figure S1). 20 g soil samples were immediately preserved on-site in RNAlater solution (Sigma-Aldrich, St. Louis, MO, United States), temporarily stored at −20°C at the Gobabeb Research and Training Centre and during transport to the laboratory, and subsequently at −80°C prior to total RNA extraction. An additional 400 g of soil for physicochemical analysis was collected in WhirlPak bags (Nasco, Fort Atkinson, WI, United States), preserved at 4°C and homogenized by sieving through a 2 mm mesh before physicochemical analyses. Soil pH, conductivity, cation exchange capacity (CEC), total nitrogen (%N), phosphorus (P), sodium (Na), potassium (K), calcium (Ca), magnesium (Mg), Chloride (Cl), Sulfate (SO_4_), ammonium (NH_4_), and nitrate (NO_3_) contents were analyzed by Bemlab (Pty) Ltd.^1^, (Strand, Western Cape, South Africa) using standard protocols.

### Total RNA Purification

Soils from 12 physicochemically similar quadrats representing all sampling times were selected for RNA extraction (Supplementary Figure S1 and Supplementary Table S2). 20 g of frozen, RNAlater-preserved soils were thawed at 4°C, centrifuged at 14,500 rpm for 5 min and supernatants were discarded. 5 volumes of ice-cold 10 mM Tris–HCl 1 mM EDTA pH 6.5 buffer containing 100 mM NaH_2_PO_4_ were added to the soil to remove RNAlater salts. The supernatant was discarded after rapid (4 min) centrifugation at 4°C. 0.5 volumes lysis buffer (5% CTAB, 0.7 M NaCl, 240 mM KH_2_PO_4_, pH 8) and an equal volume of TRI Reagent (Sigma-Aldrich) were added, and samples were vortexed at high speed for 30 s. RNA purification proceeded according to the manufacturer’s instructions. Extracted and purified total RNA was incubated with DNAseI (Invitrogen, Carlsbad, United States) for 15 min following the manufacturer’s instructions and precipitated in the presence of 20% isopropanol and 15 ng glycogen co-precipitant (GlycoBlue, Invitrogen). RNA purity and concentration were analyzed using a NanoDrop 2000 spectrophotometer (Thermo Fisher Scientific, Waltham, United States). RNA integrity and the absence of DNA contamination were confirmed in 1% agarose gel electrophoresis. The absence of RT-PCR inhibitors was tested using the Transcriptor cDNA Synthesis Kit v9 (Roche, Indianapolis, IN, United States) and universal bacterial 16S rRNA gene primers E9F (5′-GAGTTTGATCCTGGCTCAG-3′) and U1510R (5′-GGTTACCTTGTTACGACTT-3′) (Reysenbach and Pace, 1995; Hansen et al., 1998).

### Library Construction and Sequencing

One microgram DNA-free total RNA from each sample was used for each sequencing library. Due to low RNA yields, we combined RNA from two Day 2 6:00 h quadrats (Supplementary Table S1). Construction of rRNA-depleted libraries was carried out with the ScriptSeq Complete Gold Kit (Epidemiology) (Epicentre, Madison, WI, United States), following the manufacturer’s instructions. Briefly, rRNA was removed by hybridization with bead-immobilized prokaryotic and eukaryotic 28S, 23S, 18S, 16S, 5.8S, 5S, mt16S, and mt12S probes prior to RNA fragmentation and reverse transcription with tagged random hexamer primers. cDNA was amplified with TruSeq adaptors containing unique indexes (ScriptSeq Primer Set 1, Epicentre) for 15 PCR cycles. Libraries were purified using AMPure XP beads (Beckman-Coulter, Brea, United States) and final yields were measured with the High Sensitivity dsDNA reagents on a Qubit 2.0 fluorometer (Invitrogen). Multiplexed samples were quality and size analyzed in a High Sensitivity D1000 TapeStation (Agilent, Waldbronn, Germany). Libraries were single-end sequenced in a NextSeq500 v2 platform using the NextSeq 500/550 High Output v2 kit (Illumina, San Diego, United States). RNA-seq data were deposited in the ArrayExpress database ^2^ and can be accessed using the reference E-MTAB-6601.

Read quality trimming was performed using Prinseq-lite v0.20.4 (Schmieder and Edwards, 2011) on both read ends with a mean Phred value of ≥30 in a 6 base sliding window. Reads shorter than 40 bases after trimming were discarded. rRNA and human-derived reads were removed from the dataset using Bowtie2 (Langmead and Salzberg, 2012) with a database of large- and small ribosomal subunit genes from SILVA^3^, 5S rRNA genes from the 5SRNAdb repository (Szymanski et al., 2016) and the GRCh38 human genome primary assembly^4^.

### Analysis of Sequencing Reads

Functional and taxonomic profiling, and differential transcription analyses were performed using R version 3.3.3 (R Core Team, 2017). Read taxonomy was inferred from the NCBI Reference Sequence (RefSeq) database, and function was assigned based on the Kyoto Encyclopedia of Genes and Genomes (KEGG) Orthologs (KO) database (Kanehisa et al., 2016) using the MG-RAST server^5^ (Meyer et al., 2008). Read count tables were assembled and analyzed for temporal transcriptional changes using the ANOSIM permutation test with 999 permutations (Oksanen et al., 2017) and the EdgeR package (Robinson et al., 2010) for all genes with >1 count per million (cpm) in at least 3 libraries (*n* = 11). Normalized KEGG ortholog counts were fitted to a generalized log-linear model (*glmQLFit* function) (Robinson and Oshlack, 2010; McCarthy et al., 2012; Lun et al., 2016), and pairwise comparisons between all time points were performed. Additionally, grouped “day” samples from 12:00 and 18:00 h were compared to “night” samples from 24:00 and 6:00 h. KOs were considered significantly differentially expressed between time points below a false discovery rate (FDR) corrected *p*-value threshold of 0.05.

Orthologs with average log_2_CPM values >7 were used to construct a transcriptional network (Figure 4), excluding KEGG categories Human Diseases and Organismal Systems. This threshold was selected as being below the common dispersion value calculated during differential expression analysis in order to reduce interference from high-variance, low-abundance transcripts. A transcriptional network was constructed using MENA’s (Deng et al., 2012) RMT-based modeling with a correlation cutoff of 0.900 (*p* ≤ 0.005). Co-transcription was determined using Pearson’s correlation coefficients across libraries (*n* = 11). The network was visualized using Cytoscape v. 3.5.1 (Shannon et al., 2003).

## Results and Discussion

### Soil Physicochemical Characteristics

Surface soil temperatures ranged from 21.4 to 51.3°C, and soil atmospheric humidity ranged from 13 to 27.7% (Supplementary Figure S2). An average PAR flux of 1722 ± 22 μmol photons m^−2^ s^−1^ was measured at 12:00 h, but was negligible or zero at 6:00, 18:00, and 24:00 h. Consistent soil respiration was recorded throughout the experiment (Supplementary Table S2).

The physicochemistry of soil samples was globally homogeneous. Localized heterogeneity was observed in four quadrats and related mostly to salt or phosphate concentration (Supplementary Table S2 and Supplementary Figure S3).

### Library Construction and Sequence Data

A sample from each time point was selected for library construction (*n* = 12) based on homogenous soil physicochemical characteristics. Sequencing of cDNA libraries produced 285 million reads in total, with an average read length of 76 nt. After quality filtering and discarding rRNA and human-derived reads, 268 million high-quality reads were retained (Supplementary Table S3). Due to the instability of mRNA under typical desert soil conditions (as a result of oxidative damage, and radiation and desiccation-induced fragmentation (Rajeev et al., 2013), we consider that transcripts analyses provide a valid indication of recent transcriptional activity within the soil microbial population.

### Taxonomic Composition of the Active Soil Microbial Community

The taxonomic composition of the active microbial populations deduced from non-rRNA read sequences was similar throughout the study period (Supplementary Figure S4), in spite of observed variations in temperature, humidity and light (Supplementary Figure S2 and Supplementary Table S2). A single exception was the Day 2, 24:00 h library, which contained an unusually large proportion of fungal (19.5%, compared to an average of 2.8% in the remaining libraries) and Firmicutes (38.5%, compared to 5.9%) phylotypic sequences (Supplementary Figure S4). This result most likely represented random localized soil variation and not an effect of the sampling time. Fungal transcripts suggest that this could be an organic matter decomposition hotspot (Jacobson et al., 2015). This library was excluded from further analyses, as we aimed to describe diel activity in the “homogeneous” soil microbial community.

**FIGURE 1:**
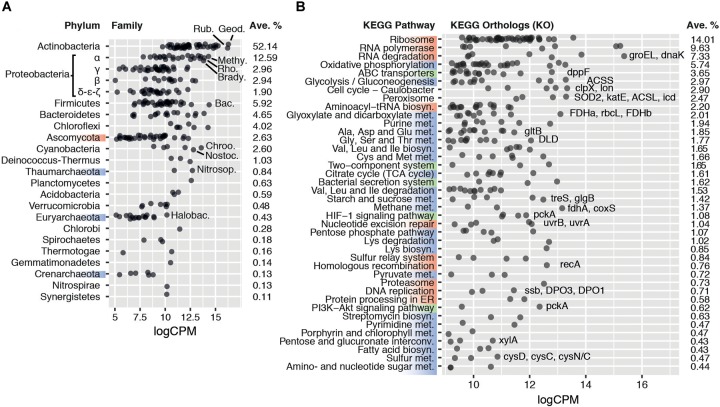
**(A)** Average transcriptional activity of the 20 most transcriptionally active microbial phyla and families. Phyla are sorted according to their average transcription levels (*aveLogCPM* function). Family transcript abundance is given in average log_2_ counts per million (logCPM). Geod.: Geodermatophilaceae; Rub.: Rubrobacteriaceae; Methy.: Methylobacteriaceae; Rho.: Rhodobacteraceae; Brady.: Bradyrhizobiaceae; Bac.: Bacillaceae; Chroo.: Chroococcales; Nostoc.: Nostocaceae; Nitrosop.: Nitrosopumilaceae; Halobac.: Halobacteriaceae. **(B)** Average transcript abundance of KEGG orthologs in the 40 most transcriptionally active KEGG pathways. Pathways are sorted according to their average log_2_ counts per million (*aveLogCPM* function). Upper KEGG classes are highlighted on the left axis by color: Genetic Information Processing (red), Metabolism (blue), and Environmental Information Processing (green). Categories Human Diseases and Organismal Systems were not included in the plot. Orthologs of particular interest are named, in order of transcript abundance, besides their respective pathway. ACSL, acyl-CoA synthetase; ACSS, acetyl-CoA synthetase; clpX, Clp protease ATP-binding subunit; *coxS*, carbon-monoxide dehydrogenase small subunit; *cysD*, sulfate adenylyltransferase subunit 2; *cysC*, adenylylsulfate kinase; cysN/C, bifunctional enzyme CysN/CysC; DLD, dihydrolipoamide dehydrogenase; *dnaK*, molecular chaperone DnaK; DPO1/3, DNA polymerase I/III; *dppF*, dipeptide transport system ATP-binding protein; FDHa/b, formate dehydrogenase alpha/beta subunit; *fdhA*, formaldehyde dehydrogenase; *glgB*, 1,4-alpha-glucan branching enzyme; *gltB*, glutamate synthase; *groEL*, chaperonin GroEL; *icd*, isocitrate dehydrogenase; *katE*, catalase; *lon*, Lon protease; *pckA*, phosphoenolpyruvate carboxykinase; *rbcL*, ribulose-bisphosphate carboxylase large chain; *recA*, recombination protein RecA; SOD2, superoxide dismutase; *ssb*, single-strand DNA-binding protein; *treS*, maltose alpha-D-glucosyltransferase/alpha-amylase; *uvrA*/*B*, excinuclease ABC; *xylA*, xylose isomerase.

The phylogenetic analysis of the remaining 11 libraries showed that members of the domain Bacteria were most active (94.2 ± 2.6% of transcripts), with Eukarya comprising 4.3 ± 1.8% and Archaea 1.5 ± 1.0%. Virus-classified reads amounted to 0.04 ± 0.02% (Figure 1A). Despite representing only a minor proportion, this is, to our knowledge, the first report of transcriptionally active viruses in dessicated hot desert soils, where lysogeny is considered to be the dominant state of virus populations (Zablocki et al., 2015). However, the read volume was insufficient to provide a comprehensive survey of transcribed viral genes.

Seven bacterial phyla (Actinobacteria, Proteobacteria, Firmicutes, Bacteroidetes, Chloroflexi, Cyanobacteria, and Deinococcus-Thermus) and one eukaryal phylum (Ascomycota) each contributed more than 1% of the classified reads, jointly comprising 93.1 ± 2.3% of the total active community (Figure 1A and Supplementary Figure S4). The most active phylum globally was Actinobacteria, producing 52.1 ± 5.4% of the classified transcripts, followed by Proteobacteria, with 20.2 ± 2.6% of the transcripts. Two prominent actinobacterial families, Geodermatophilaceae and Rubrobacteraceae, represented 8.2 ± 1.8% and 7.0 ± 1.9%, respectively (Figure 1A). Both families have been routinely detected in desert soils and their members typically exhibit high stress tolerance and are metabolically versatile (Rainey et al., 2005; Favet et al., 2013; Albuquerque and da Costa, 2014; Normand et al., 2015; Sghaier et al., 2016).

### Functional Profile of the Microbial Community

All core metabolic pathways were transcribed (Figure 1B), including replication genes, indicating that the active fraction of the soil microbial community had complete functionality. This transcriptional profile strongly suggests that the sequenced mRNA did not originate from spores or other dormant forms, which have been shown to only accumulate selected transcripts related to resuscitation processes for a period after the onset of dormancy (Segev et al., 2012; Rajeev et al., 2013).

The transcriptional profiles strongly suggest the existence of a xero resistant microbial community in this hyperarid desert soil niche. Tolerance; i.e., survival with impaired or no activity and no growth, is regarded as the most common strategy adopted by microbial communities under extreme xeric stress, such as in hyperarid desert soils (Lebre et al., 2017). Active microbial populations have recently been detected in hyperarid soils: from the Atacama Desert by measuring ATP (Schulze-Makuch et al., 2018) and in Namib Desert gravel plain soils from the determination of respiration rates (Armstrong et al., 2016). Our transcription results, which demonstrate that at least a fraction of the microbial community is functional, suggest that resistance, rather than tolerance, is a strategy adopted by some of the resident taxa. Resistance is here defined as the maintenance of function, despite the impositions of extreme environmental parameters (i.e., hyperaridity) (Harrison et al., 2007).

Genes encoding elements of stress resistance and damage repair mechanisms were highly transcribed. Chaperone genes *groEL* and *dnaK* (4.9% and 1.5% of the classified transcripts, respectively) (Figure 1B), and protease genes involved in protein quality control (e.g., *clpX/P* and *lon*; 1.7% and 0.7%, respectively) were among the most transcribed. Furthermore, the high relative abundances of peroxisomal orthologs (2.4%), such as superoxide dismutase (SOD) and catalase (*katE*), as well as DNA repair gene transcripts (*recA*, *uvr*, Figure 1B), support the widely held view that radiation- and desiccation-induced damage (particularly related to oxidation processes) are the major stresses for microbial cells in hyperarid hot desert soils (Makhalanyane et al., 2015). The production of compatible solutes and capsule formation, which are common microbial adaptation mechanisms for desiccation tolerance (Lebre et al., 2017), were suggested by polysaccharide and trehalose biosynthesis gene transcripts such as the alpha-glucan branching enzyme gene *glgB* (0.4% of transcripts) (Rashid et al., 2016; Figure 1B). Overall, the transcriptional profile of the microbial community coherently reflects known strategies of desiccation resistance predicted from genomic analyses of desiccation-tolerant microorganisms (Lebre et al., 2017; Schulze-Makuch et al., 2018). Furthermore, given the transcription of most core metabolic pathways (particularly, of growth-related gene transcripts), we conclude that a fully active population remains during periods of hyperaridity.

### Nutrient Cycling and Key Active Taxa

Carbon, nitrogen, and phosphorus are the major limiting nutrients for soil microbial communities, and for oligotrophic desert soil communities in particular (Cleveland and Liptzin, 2007; Delgado-Baquerizo et al., 2013; Johnson et al., 2017). The transcriptional activity of orthologs involved in nitrogen species reduction to assimilable ammonia, phosphorus import in organic or inorganic forms, sulfur reduction, and inorganic carbon fixation in the community was examined in further detail.

**FIGURE 2:**
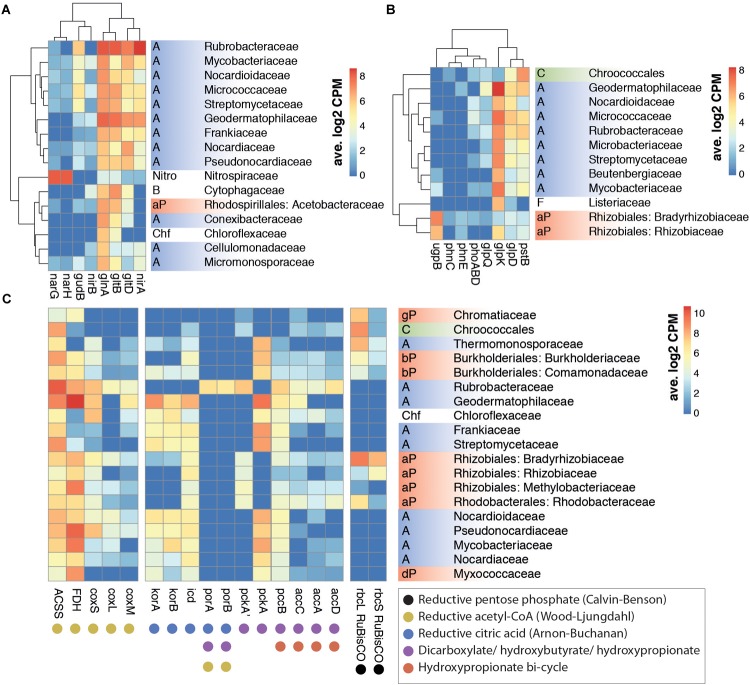
Correlation between family level taxonomy and key KEGG ortholog transcription from nitrogen **(A)**, phosphorus **(B)**, and carbon (C) assimilation pathways in selected prokaryotic taxa. **(A)** Nitrogen metabolism orthologs: *gln*, glutamine synthetase; *glt*, glutamate synthase; *gud*, glutamate dehydrogenase; *nar*, nitrate reductase; *nir*, nitrite reductase. **(B)** Phosphorus assimilation and G3P metabolism orthologs: *glpD*, glycerol 3-phosphate dehydrogenase; *glpK*, glycerol kinase; *glpQ*, glycerophosphodiester phosphodiesterase; *phn*, phosphonate transport; *phoABD*, alkaline phosphatase; *pst*, phosphate transport; *ugp*, sn-glycerol-3-phosphate transport. **(C)** Carboxylase gene orthologs involved in carbon fixation pathways: ACSS, acetyl-CoA synthase; *acc*, acetyl-CoA carboxylase; FDH, formate dehydrogenase; *icd*, isocitrate dehydrogenase; *kor*, 2-oxoglutarate synthase; *cox*, CO dehydrogenase; *pcc*, acetyl/propionyl-CoA carboxylase; *pck*, PEP carboxylase; *por*, pyruvate synthase; *rbc*, RuBisCO. Phylum abbreviations: A: Actinobacteria; B: Bacteroidetes; C: Cyanobacteria; Chf: Chloroflexi; F: Firmicutes; Nitro: Nitrospirae; P: Proteobacteria (a: alpha, b: beta, d: delta, g: gamma). Only families with >6 average log_2_CPM for the selected genes were included in **A** and **B**. Hierarchical clustering of rows and columns was performed with *hclust* function.

**FIGURE 3:**
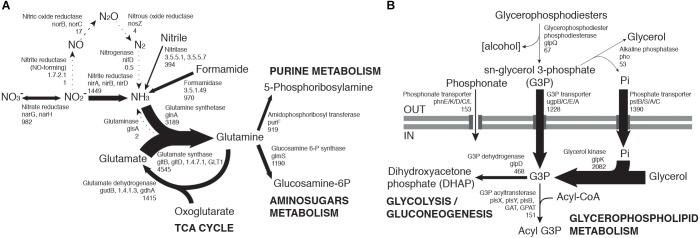
Community-level transcription of nitrogen **(A)** and phosphorus **(B)** assimilation pathways, including glycerol phosphate metabolism. Gene codes in bold highlight the most abundant orthologs from a group performing the same function, when there is a large transcript abundance difference. Numbers below gene codes show the total average counts per million (cpm) of all orthologs. Arrow thickness in each figure is proportional to the indicated cpm value.

High levels of functional redundancy were evident (Figure 2). However, some important ecosystem functions appeared to be taxon-specific. For example, nitrate reductase (*nar*) genes, which encode key enzymes in nitrogen assimilation in soils (Merrick and Edwards, 1995; Figure 3A), were transcribed almost exclusively by members of the Nitrospiraceae family (Figure 2A), indicating that this family plays a key role in the nitrogen cycling of Namib desert soil communities.

### Nitrogen Assimilation

Nitrogen-fixing bacterial taxa such as Geodermatophilaceae, Frankiaceae and Rhizobiales (Merrick and Edwards, 1995; Sellstedt and Richau, 2013) were among the most active taxa (Figure 1A). However, transcripts relating to the nitrogen metabolism KEGG pathway represented a small portion (0.2%) of our soil metatranscriptomes and virtually no *nifD* nitrogenase transcripts were detected (Figure 3A). These findings are compatible with recent observations that hypolithic communities, and not surface soil communities, were the primary sources of N_2_-fixation in Namib Desert gravel plains (Ramond et al., 2018).

Transcriptome data suggested that nitrate reduction, most transcribed by the Nitrospiraceae family, and nitrite reduction, primarily transcribed in actinobacterial taxa (*nar* and *nir* genes, respectively, Figure 2A), were the dominant processes in the generation of biologically available nitrogen in the community from a NO_3_^−^ and NO_2_^−^ reservoir (Figure 3A). These nitrogen species may be accumulated in soils during infrequent wet periods, possibly as a result of the activation of genes and microorganisms inhibited during desiccated conditions (Scherer et al., 1984), or from atmospheric deposition processes (Báez et al., 2007; Jia et al., 2016).

### Phosphorus and Sulfur Assimilation

Most phosphorus is available to soil microbial communities as inorganic phosphate (Pi), solubilized from the mineral soil fraction or released from organic molecules by the alkaline phosphatase (White and Metcalf, 2007). *Pst* phosphate transporter gene transcripts were abundant in the community (1390 average counts per million, cpm). Organic phosphate sources were also possibly exploited, as suggested by transcription of the *phn* phosphonate transporter gene (153 cpm) and especially the sn-glycerol 3-phosphate (G3P) transporter gene *ugp* (1228 cpm) (Figure 3B). Although the expression of *phn* and *ugp* can be inhibited by Pi (Schowanek and Verstraete, 1990; Brzoska et al., 1994), organic P utilization may still be an important microbial community trait in oligotrophic desert environments (Vikram et al., 2016). The *ugp* genes were principally transcribed by members of the Order Rhizobiales (Class alpha-Proteobacteria), potentially replacing Pi transport as a phosphorus acquisition mechanism (Figure 2B). Plant exudates or membrane phospholipids are possible sources of G3P in soils (Ding et al., 2012; Collins et al., 2014; Lidbury et al., 2017). The *glpQ* gene product can cleave these compounds, releasing G3P and triggering activation of the *ugp* transporter genes (Brzoska and Boos, 1988). *GlpQ* is an extracellular enzyme which has been implicated in cooperative interactions between proteobacteria (Lidbury et al., 2017). In our dataset, *glpQ* was mostly transcribed in Actinobacteria. The most active actinobacterial family Geodermatophilaceae, however, transcribed *glpQ*, but not the G3P transporter *glp* or the alkaline phosphatase *pho* genes, which would dephosphorylate G3P, releasing Pi for its own consumption (Figure 2B). Our data therefore suggest a putative interaction between Geodermatophilaceae and Rhizobiales, with the former providing access to phosphorus as G3P for the latter. This interaction may be of considerable importance for desert community maintenance, as both taxa were amongst the most transcriptionally active (8.2% and 6.1%, respectively) (Figure 1A).

Reductive sulfate assimilation (*cys* genes) was the dominant transcribed S-cycling pathway in the community. However, transcripts for the sulfate transporter *cysPUWA* were mostly associated to proteobacteria, particularly the Burkholderiales family, suggesting a central role of this group in sulfur assimilation and cycling.

### Carbon Fixation

Defining features of arid soils are low productivity and low organic carbon content (Delgado-Baquerizo et al., 2013). Hyperaridity imposes severe constraints on oxygenic photosynthesis, for which water is the electron donor (Warren-Rhodes et al., 2006). Furthermore, soil communities outside of sheltered fertile islands (i.e., hypoliths, endoliths, or biological soil crusts) typically have a very low abundance of phototrophic cyanobacteria (Makhalanyane et al., 2013; Stomeo et al., 2013). Perhaps not surprisingly, transcription of photosynthetic pathway genes and phototrophic organisms was limited in our sequence dataset (Figure 1B,A). Notably, reads classified within the Glyoxylate and Dicarboxylate pathway (2.0%) exceeded those assigned to photosynthetic KEGG pathways and, surprisingly, also significantly exceeded those from the TCA cycle (0.3% and 1.6%, respectively, two-tailed *t*-test *p* < 0.005) (Figure 1B). We also observed a higher number of transcripts assigned to acetyl-CoA synthetase (ACSS, 9874 cpm) and formate dehydrogenase (FDH, 10254 cpm) compared to RuBisCO (*rbcL*/*S*, 2672 cpm) (Figure 1B). These observations strongly suggest that chemoautotrophic (“dark”) carbon fixation and/or CO_2_ reassimilation mechanisms are important microbial processes by which inorganic C enters the soil microbial community. Chemoautotrophic carbon fixation has been shown as an important process in marine environments, even when photosynthesis is active (Palovaara et al., 2014; Aylward et al., 2015), and is potentially a major process in soils (King and Weber, 2007; Pratscher et al., 2011). We therefore examined the activity of carboxylase genes, and their distribution between different families of microorganisms, in greater depth.

Although RuBisCO gene transcripts (*rbc*) from the Calvin-Benson-Bassham (CBB) cycle were significant (average 2672 cpm) (Figure 1B), the majority were assigned to non-photosynthesizing alpha-Proteobacteria rather than to Cyanobacteria (Figure 2C). This suggested that the CBB cycle acted predominantly in chemoautotrophic CO_2_ fixation or as an electron sink (Badger and Bek, 2008; McKinlay and Harwood, 2010), rather than in photosynthesis.

Orthologs of the acetyl-CoA synthase ACSS (9874 cpm), CO dehydrogenase *coxS* (1701 cpm) and formate dehydrogenase FDH (10254 cpm) genes, involved in the reductive acetyl-CoA cycle (Wood-Ljungdahl pathway), were significantly transcribed in a wide range of taxa (Figure 2C). These carboxylases are widely distributed in soil bacteria (King and Weber, 2007) and are active in desert actinobacteria (Sghaier et al., 2016). The activity of these genes in Namib Desert soil microbial communities may be related to the very low energy requirements and the capacity to coassimilate one-carbon compounds or acetate of this pathway (Fuchs, 2011), making it well suited to oligotrophic niches.

The reductive citric acid cycle (Arnon-Buchanan cycle) carboxylases *kor* (2-oxoglutarate synthase) and *icd* (isocitrate dehydrogenase) were transcribed by many actinobacterial families, but surprisingly not by Rubrobacteraceae (Figure 2C). Instead, Rubrobacteraceae transcribed the acetyl and propionyl-CoA carboxylase *pccB* and *accA/C/D*, the phosphoenolpyruvate (PEP) carboxylase *pckA* and the pyruvate synthase *por*, which participate in other pathways of chemoautotrophic carbon fixation (Fuchs, 2011; Figure 2C). These pathways could only be partialy detected in Rubrobacteraceae, as transcripts for several key genes (e.g., malonyl-CoA reductase, 4-hydroxybutyryl-CoA dehydratase) were not identified. These pathways, either full or partial, allow prokaryotes to coassimilate reduced and uncommon C compounds and to fix carbonate (Fuchs, 2011; Zarzycki and Fuchs, 2011). Our results therefore suggest that the actinobacterial Rubrobacteraceae family may be important in inorganic carbon acquisition in desert soils, partly due to a high plasticity in chemoautotrophic metabolism.

### Circadian Differential Gene Expression

Environmental variations often cause microbial communities to exhibit differential activity profiles over temporal timescales (e.g., daily or seasonally), both in phototrophic and non-phototrophic groups (van der Meer et al., 2005; Klatt et al., 2013; Ottesen et al., 2013, 2014; Aylward et al., 2015). An initial permutation test on sample group dissimilarities was performed using ANOSIM (Oksanen et al., 2017). The analysis showed a low but significant dissimilarity between “day” (12:00 and 18:00) and “night” (24:00 and 6:00) groups (*R* = 0.2053; *p* = 0.018, 999 permutations). These results are consistent with the hypothesis that diurnal and reciprocal variations in soil temperature and humidity (Supplementary Figure S2) are significant drivers of gene transcription in the microbial community.

Diel transcriptional periodicity was also examined at the gene ortholog level using EdgeR (Robinson et al., 2010). Time pairs were contrasted independently, as well as “day” vs. “night” groups as defined above. Interestingly, pairwise comparisons identified no differentially expressed orthologs (*p* > 0.05) between the 12:00 and 18:00 or between the 24:00 and 6:00 datasets. When “day” and “night” data were contrasted, 13 of 2265 orthologs (0.57%) were significantly (*p* < 0.05) induced during the night (Supplementary Table S4). None were highly transcribed orthologs, suggesting that under extreme dry conditions, desert soil communities are generally functionally stable and that their principal functions are not strongly regulated on a diel scale.

This conclusion has implications in terms of the perceived drivers of microbial community function, as our results suggest that the constant xeric stress is a more significant driver of *in situ* functionality than daily environmental variations (temperature, soil atmospheric moisture or light). We predict that this functional stability would only be substantially disrupted by stochastic events such as rainfall, which is recognized as a main driver of community assembly and activity in arid soil environments (Belnap et al., 2005; Pointing and Belnap, 2012; Frossard et al., 2015; Armstrong et al., 2016; Scola et al., 2017).

Surprisingly, we observed a marked enrichment in differentially transcribed eukaryal orthologs, including tubulin, dynein, myosin, SF3B, *dnaJ*, and ANP1 genes (Supplementary Table S4). This suggests that the active fungi, which only represented 2.8% of the total transcripts, were most active during the cooler and higher atmospheric humidity night hours. This functional behavior contrasts with the stable activity pattern observed for the rest of the community, but is consistent with previous observations for fungi and lichens from arid environments, where these taxa appear to grow optimally during small air moisture pulses (Palmer et al., 1987; Jacobson et al., 2015).

### Transcriptional Network Analysis

**FIGURE 4:**
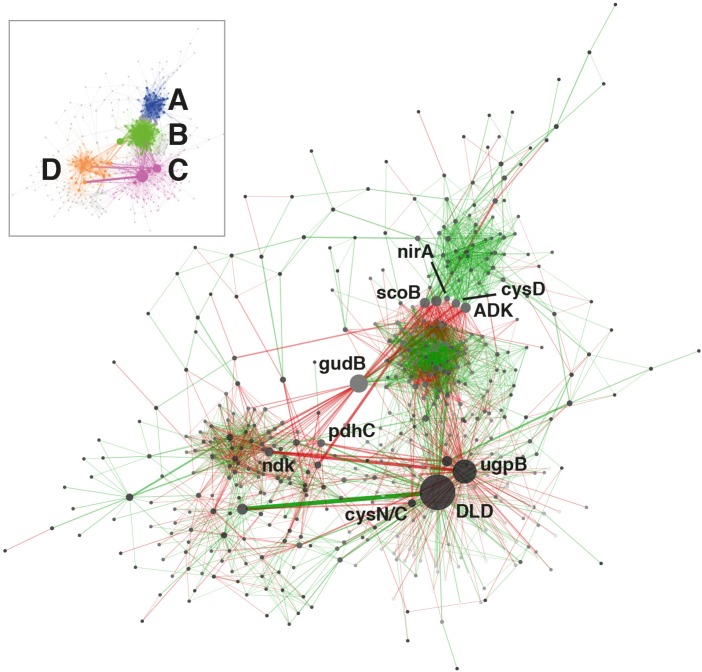
KEGG ortholog transcriptional network. Only orthologs with average abundances of log_2_ cpm >7 in the complete dataset were used for computation. Node size is proportional to betweenness centrality and edge thickness is proportional to betweenness. Green or red edge lines indicate a shared positive or negative correlation, respectively. Ortholog abbreviations: ADK, adenosine kinase; *cysD*, sulfate adenylyltransferase (sulfate-activating complex); *cysN/C*, bifunctional enzyme *CysN*/*CysC* (sulfate-activating complex); DLD, dihydrolipoamide dehydrogenase; *gudB*, glutamate dehydrogenase; *ndk*, nucleoside-diphosphate kinase; *nirA*, ferredoxin-nitrite reductase; *pdhC*, pyruvate dehydrogenase; scoB, 3-oxoacid CoA-transferase; *ugpB*, sn-glycerol 3-phosphate transport system.

The temporal co-variation of KOs was determined in order to examine whether coordinated patterns of gene transcription existed within the soil community. 624 orthologs were used to construct a transcriptional network, 83.3% of which (520) clustered into 4 distinct modules (A to D, Figure 4). The larger clusters A and B were composed of positively interrelated orthologs, although no specific functional enrichment within each module was observed.

The principal transcriptional network clusters were connected through three orthologs: the dihydrolipoamide dehydrogenase DLD, the G3P transporter subunit *ugpB* and the glutamate dehydrogenase gene *gudB*. These genes occupy network hub positions and changes in their transcriptional status could result in large shifts in community function. The dominant metabolic function associated with the highly transcribed (3917 cpm, Figure 1B) DLD gene is associated with the TCA cycle, but this gene has also been shown to affect sugar transport and capsule formation via direct interactions with membrane transporters (Tyx et al., 2011). The importance of exopolysaccharides in desiccation resistance (Lebre et al., 2017), and the TCA cycle in carbon metabolism regulation, support the centrality of DLD in the community network. The gene *ugpB*, as previously discussed, was linked to the rhizobial community as part of a nearly exclusive phosphorus assimilation mechanism (Figure 2B). The *gudB* gene product catalyzes the synthesis of glutamate, the principal acceptor metabolite in NH_3_ assimilation (Merrick and Edwards, 1995; Figure 3A).

The network was also characterized by a group of orthologs connecting the main clusters A and B. Three of these are involved in nitrogen (*nirA*) (Merrick and Edwards, 1995; Figure 3A), sulfur (*cysD*) (Pinto et al., 2004) and central carbon metabolism (*scoB*) (Corthésy-Theulaz et al., 1997; Figure 1B).

Globally, network analysis suggested that transcriptional activity of the community was structured around a selection of hub genes involved in the central steps of nitrogen (*nir*A, *gudB*) (Figure 3A) and sulfate assimilation (*cysD*, *cysN/C*), phosphorus acquisition (*ugpB*) (Figure 3B) and carbohydrate metabolism (DLD, *scoB*). The network analysis did not significantly implicate genes related to environmental stress resistance and damage repair (e.g., chaperones, proteases, SOD, *uvr*, *rec*), despite these genes being consistently transcribed (Figure 1).

## Conclusion

It is widely accepted that the extreme conditions in hot desert open soils limit both microbial and plant life (Pointing and Belnap, 2012; Makhalanyane et al., 2015) and that microbial activity is spatially fragmented, temporally limited and water-driven (Belnap et al., 2005; Pointing and Belnap, 2012; Collins et al., 2014). We have demonstrated that a diverse and consistently active edaphic microbial community exists in hyperarid Namib Desert soils, and that the “active” community is dominated by non-photosynthetic bacteria. Transcripts from all central metabolic pathway genes were detected, suggesting consistent transcriptional activity during the study period. We therefore suggest that desiccation-resistant microbial subpopulations remain active and able to proliferate during dry periods, rather than surviving in inactive states (Harrison et al., 2007). Despite the observation of regular environmental fluctuations, only moderate diel changes were observed in prokaryotic transcriptional activity, although a significant activation of fungal genes was noted during the night hours.

Our results highlight the importance of nutrient acquisition for the maintenance of a potentially active microbial population in hyperarid desert soils. However, in contrast to expectations for this extreme environment, stress related gene transcripts were not core elements of functional assemblies, despite their known importance for microbial survival.

Overall, our results show that mRNA transcript analyses can provide valuable information on potential community functionality in dry soil habitats and suggest that these communities may be important in local biogeochemical cycling. This approach is not only more detailed but also potentially offers a better estimation of microbial activity states than rRNA amplicon sequencing (Blazewicz et al., 2013). Future research should be directed toward understanding the kinetics of microbial processes, and particularly carbon fixation, during dry periods in desert soil ecosystems.

## Author’s Note

This work has been made available as a preprint ahead of publication in the bioRxiv repository (León-Sobrino et al., 2018) and was assigned the doi: 10.1101/432427 (see References).

## Author Contributions

CL-S, J-BR, and DC conceived the experiment and participated in the interpretation of results and writing of the manuscript. GM-K provided logistical support and field advice in the Namib Desert. CL-S performed all experimental work and bioinformatic analysis of the sequencing output. DC provided the funding.

## Conflict of Interest Statement

The authors declare that the research was conducted in the absence of any commercial or financial relationships that could be construed as a potential conflict of interest.

## Footnotes

1 http://www.bemlab.co.za/

2 www.ebi.ac.uk/arrayexpress

3 https://www.arb-silva.de/

4 ftp://ftp.ncbi.nlm.nih.gov/genomes/archive/old_genbank/Eukaryotes/vertebrates_mammals/Homo_sapiens/GRCh38/seqs_for_alignment_pipelines/GCA_000001405.15_GRCh38_no_alt_analysis_set.fna.bowtie_index.tar.gz

5 http://metagenomics.anl.gov/
