# Supplementary material for: Nutrient Acquisition, Rather Than Stress Response Over Diel Cycles, Drives Microbial Transcription in a Hyper-Arid Namib Desert Soil
Source: Front Microbiol. 2019 May 14;10:1054. doi: 10.3389/fmicb.2019.01054 (PMC6527771; doi:10.3389/fmicb.2019.01054)
Supplement: Supplementary file 1 [file Data_Sheet_1.doc]

## Supplementary Materials: Nutrient acquisition, rather than stress response over diel cycles, drives microbial transcription in hyperarid hot desert soils

Carlos León-Sobrino, Jean-Baptiste Ramond, Gillian Maggs-Kölling and Don A Cowan

**Supplementary Figure S1**: Sampling site description.

**Supplementary Figure S2**: Environmental conditions during the experiment.

**Supplementary Figure S3**: Physicochemical analysis biplot of sampled soils.

**Supplementary Figure S4**: Relative abundance of phyla in sequences from the cDNA libraries.

**Supplementary Table S1**: Local climatic conditions prior to the experiment.

**Supplementary Table S2**: Physicochemical analysis and environmental condition measurements of sampled soils.

**Supplementary Table S3**: Library sequence outputs and mapping statistics.

**Supplementary Table S4**: Significantly differentially transcribed KEGG orthologs between grouped day and night metatranscriptome libraries.

## Supplementary figures

Figure S1: Distribution of the quadrats sampled within the sampling plot. Prefix numbers indicate day of sampling, suffix the hour, and the middle letters identify each of the three sectors sampled at each time (A, B, and C). Sectors selected for RNA library construction are marked in red letters. Circles show the position of CO_2_ an photosynthetically active radiation (PAR) flux measurements, and stars the position of iButtons for temperature and relative humidity monitoring. Environmental and soil physicochemical analysis results are given in Figure S2 and Table S1.


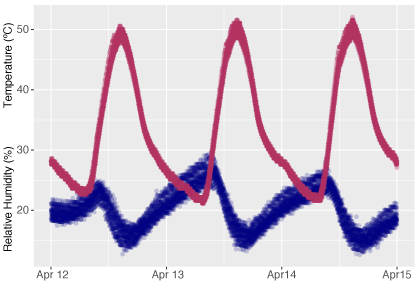


Figure S2: Temperature (maroon) and relative humidity (blue) records during the study period.

Figure S3: Principal component analysis (PCA) biplot of soil physicochemical characteristics from all sampled sectors (see Figure S1 and Table S1). Each measured variable values were scaled prior to the analysis.


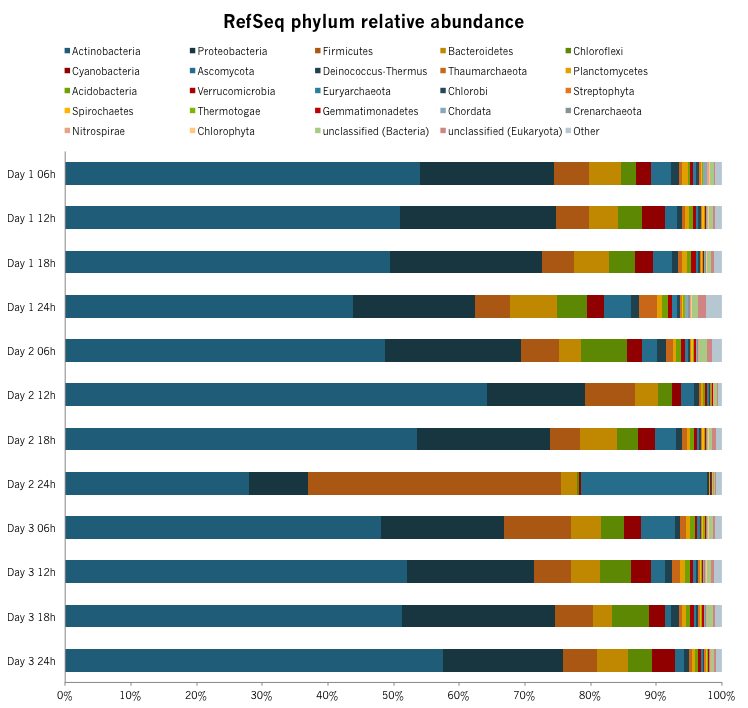


Figure S4: Relative abundance of phyla in sequences from the cDNA libraries. Reads were classified according to RefSeq hits and their associated taxonomic affiliation, with a significance e-value threshold of 10^-5^. Phyla with mean relative abundance below 1% were grouped in the "Other" category.

## Supplementary tables

| Month Year | Air temp. (avg) | Air temp. (min) | Air temp. (max) | Soil temp. (avg) | Precip. (total) | Wind Speed (vc avg) | Wind direction (vc avg) | Wind Speed (max) | Max. speed wind dir. | Humidity |
| --- | --- | --- | --- | --- | --- | --- | --- | --- | --- | --- |
|  | [°C] | [°C] | [°C] | [°C] | [mm] | [m/s] | [deg.] | [m/s] | [deg.] | [%] |
| oct-14 | 17.8 | 6.4 | 34.2 | 26.0 | 0.0 | 1.6 | 267 | 8.1 | 192 | 60.5 |
| nov-14 | 21.3 | 8.8 | 40.8 | 29.2 | 8.6 | 1.5 | 250 | 10.0 | 190 | 49.1 |
| dic-14 | 20.3 | 10.0 | 35.1 | 30.1 | 0.1 | 2.2 | 280 | 8.8 | 214 | 58.1 |
| ene-15 | 22.2 | 12.2 | 37.7 | 31.0 | 6.0 | 2.3 | 287 | 8.9 | 333 | 64.2 |
| feb-15 | 21.0 | 10.6 | 34.2 | 29.5 | 0.0 | 1.8 | 275 | 8.0 | 202 | 57.0 |
| mar-15 | 24.1 | -0.6 | 41.1 | 30.9 | 0.9 | 1.4 | 246 | 9.0 | 193 | 44.1 |
| abr-15 | 21.8 | 6.6 | 37.3 | 27.8 | 0.0 | 0.9 | 266 | 8.7 | 59 | 47.7 |
| may-15 | 23.6 | 9.5 | 37.6 | 26.4 | 0.0 | 0.9 | 156 | 13.0 | 19 | 26.5 |
| jun-15 | 16.9 | 3.1 | 33.5 | 20.0 | 0.0 | 1.1 | 138 | 10.7 | 58 | 41.1 |
| jul-15 | 15.9 | 1.6 | 34.8 | 19.5 | 0.0 | 0.7 | 123 | 12.8 | 64 | 47.7 |
| ago-15 | 17.0 | 4.3 | 35.4 | 21.1 | 0.1 | 0.2 | 200 | 12.6 | 66 | 51.9 |
| sep-15 | 17.7 | 5.3 | 39.2 | 23.8 | 0.0 | 0.9 | 229 | 11.5 | 79 | 54.9 |
| oct-15 | 19.9 | 6.7 | 38.0 | 27.2 | 0.0 | 1.6 | 263 | 12.1 | 193 | 54.4 |
| nov-15 | 20.8 | 9.4 | 37.9 | 29.2 | 1.3 | 1.6 | 262 | 16.5 | 77 | 50.0 |
| dic-15 | 23.7 | 11.7 | 38.4 | 32.1 | 0.1 | 1.9 | 276 | 9.5 | 326 | 52.5 |
| ene-16 | 24.3 | 13.4 | 42.3 | 33.4 | 0.2 | 2.0 | 274 | 8.4 | 190 | 58.3 |
| feb-16 | 23.2 | 13.3 | 36.3 | 31.8 | 0.1 | 2.0 | 272 | 7.3 | 199 | 58.1 |
| mar-16 | 22.2 | 8.8 | 38.7 | 30.1 | 0.0 | 1.3 | 262 | 12.0 | 62 | 53.1 |
| abr-16 | 25.5 | 10.3 | 39.5 | 29.9 | 0.0 | 0.8 | 207 | 10.2 | 331 | 37.1 |
| Days with precipitation ≥1 mm | | | | | | | | | | |
| 16-nov-14 | 24.0 | 14.0 | 36.9 | 30.9 | 8.1 | 1.9 | 263 | 7.3 | 204 | 39.4 |
| 25-ene-15 | 24.4 | 20.7 | 31.6 | 30.0 | 3.1 | 1.2 | 163 | 6.7 | 251 | 70.5 |
| 26-ene-15 | 23.7 | 20.1 | 31.4 | 30.7 | 2.8 | 2.0 | 293 | 6.4 | 265 | 75.2 |
| 25-nov-15 | 25.8 | 15.5 | 35.8 | 32.8 | 1.0 | 2.2 | 218 | 7.4 | 200 | 24.1 |

Table S1: Monthly weather records up to 18 months prior to the experimental dates. Lower subtable shows daily weather records for any day where a precipitation exceeding 1 mm was recorded. Data collected by the Gobabeb Meteorological Station (23°33'38"S 15°02'28"E) and obtained from SASSCAL (www.sasscalweathernet.org; ID: 8893).

| ID | Time  (day hour) | pH | EC  mS/m | P  mg/l | Na  mg/l | K  mg/l | Ca  mg/l | Mg  mg/l | Cl  mg/l | SO_4_  mg/l | NH_4_-N  mg/l | NO_3_-N  mg/l | N  % | CEC  (pH 7) cmol(+)/kg | T  ºC | RH  % | CO_2_  µmol/mol  (30 s) | PAR  µmol photons m^-1^ s^-1^ |
| --- | --- | --- | --- | --- | --- | --- | --- | --- | --- | --- | --- | --- | --- | --- | --- | --- | --- | --- |
|  |  |  |  |  |  |  |  |  |  |  |  |  |  |  |  |  |  |  |
| 1A6 | d1 06:00 h | 6.58 | 22 | 0.54 | 23.16 | 8.63 | 17.81 | 3.87 | 31.67 | 41.7 | 0.73 | 1.11 | 0.04 | 3.38 | 23.4 | 26.1 | 133.8 | 3.2 |
| **1B6** |  | 6.69 | 22 | 0.61 | 26.44 | 8.6 | 19.18 | 3.50 | 30.95 | 51.95 | 0.63 | 1 | 0.05 | 3.22 |  |  |  |  |
| 1C6 |  | 6.79 | 56 | 2220 | 102.19 | 18.50 | 29.51 | 4.35 | 99.34 | 151.54 | 1 | 1.18 | 0.05 | 3.01 |  |  |  |  |
| 1A12 | d1 12:00 h | 7.02 | 21 | 0.83 | 18.25 | 6.85 | 18.89 | 3.42 | 29.87 | 34.64 | 0.74 | 1.13 | 0.05 | 3.13 | 42.8 | 12.7 | 136.8 | 1740.8 |
| **1B12** |  | 6.97 | 18 | 0.91 | 17.28 | 6.85 | 16.04 | 3.87 | 30.95 | 28.3 | 0.69 | 1.04 | 0.05 | 2.75 |  |  |  |  |
| 1C12 |  | 6.91 | 19 | 0.81 | 22.83 | 8.57 | 14.36 | 4.36 | 26.27 | 33.61 | 0.71 | 0.88 | 0.05 | - |  |  |  |  |
| **1A18** | d1 18:00 h | 6.83 | 21 | 0.88 | 22.61 | 13.35 | 14.06 | 3.11 | 38.51 | 30.5 | 0.81 | 1.37 | 0.05 | 3.1 | 38.9 | 21.3 | 140.6 | 10.3 |
| 1B18 |  | 6.89 | 184 | 2788 | 52.75 | 12.13 | 17.47 | 3.38 | 6795.27 | 34.21 | 1.18 | 2.03 | 0.05 | 3.86 |  |  |  |  |
| 1C18 |  | 7.05 | 49 | 0.54 | 19.22 | 6.82 | 17.40 | 2.80 | 106.9 | 34.97 | 0.59 | 1.06 | 0.06 | 3.07 |  |  |  |  |
| 1A24 | d1 24:00 h | 7.08 | 118 | 0.32 | 90.17 | 12.70 | 16.63 | 3.13 | 121.65 | 68.85 | 1.96 | 1.36 | 0.04 | - | 26.6 | 28.3 | 133.6 | 0 |
| 1B24 |  | 7.39 | 29 | 0.15 | 259.08 | 23.36 | 30.68 | 3.04 | 52.19 | 156.47 | 0.53 | 1.76 | 0.05 | - |  |  |  |  |
| **1C24** |  | 7.21 | 22 | 0.59 | 29.97 | 9.58 | 18.23 | 3.31 | 41.39 | 39.11 | 1.25 | 1.33 | 0.05 | - |  |  |  |  |
| **2A6** | d2 06:00 h | 7.1 | 29 | 0.47 | 24.24 | 7.15 | 18.65 | 3.05 | 30.59 | 49.09 | 0.68 | 0.97 | 0.05 | 3.81 | 22.4 | 34.7 | 133.3 | 1.1 |
| **2B6** |  | 7.19 | 21 | 0.38 | 38.85 | 12.29 | 19.20 | 2.30 | 26.99 | 85.65 | 0.67 | 0.94 | 0.06 | 3.62 |  |  |  |  |
| 2C6 |  | 6.54 | 27 | 0.50 | 22.77 | 7.38 | 16.10 | 2.74 | 30.59 | 39.88 | 0.82 | 0.99 | 0.06 | - |  |  |  |  |
| 2A12 | d2 12:00 h | 7.35 | 46 | 0.13 | 600.68 | 38.79 | 79.14 | 11.67 | 142.17 | 93.58 | 1 | 6.58 | 0.05 | - | 43.3 | 12.9 | 136.3 | 1697.5 |
| **2B12** |  | 7.28 | 30 | 0.8 | 48.85 | 19.18 | 16.11 | 2.87 | 68.02 | 53.97 | 0.83 | 1.84 | 0.05 | 3.4 |  |  |  |  |
| 2C12 |  | 6.89 | 23 | 0.68 | 34.31 | 14.32 | 19.79 | 2.92 | 42.11 | 56.09 | 0.72 | 1.32 | 0.05 | 3.46 |  |  |  |  |
| **2A18** | d2 18:00 h | 6.98 | 21 | 0.55 | 24.36 | 10.10 | 18.60 | 3.10 | 29.15 | 48.02 | 0.69 | 1.19 | 0.05 | 3.79 | 39.9 | 20.4 | 137.0 | 4.1 |
| 2C18 |  | 6.71 | 61 | 0.61 | 27.75 | 8.18 | 13.89 | 3.59 | 29.51 | 33.22 | 0.77 | 1.14 | 0.05 | 3.46 |  |  |  |  |
| **2A24** | d2 24:00 h | 7.05 | 35 | 0.93 | 85.61 | 34.43 | 46.30 | 5.07 | 137.49 | 111.52 | 0.99 | 1.85 | 0.05 | - | 27.9 | 21.7 | 140.6 | 0 |
| 2B24 |  | 6.7 | 28 | 0.85 | 48.09 | 17.84 | 13.61 | 3.07 | 58.31 | 53.5 | 1.71 | 2.84 | 0.04 | 3.33 |  |  |  |  |
| 2C24 |  | 6.4 | 84 | 0.68 | 29.77 | 10.43 | 22.09 | 2.73 | 50.39 | 40.75 | 0.91 | 1.32 | 0.05 | - |  |  |  |  |
| 3A6 | d3 06:00 h | 6.75 | 105 | 0.33 | 173.77 | 24.77 | 19.58 | 3.32 | 250.86 | 78.92 | 0.56 | 3.41 | 0.05 | 3.59 | 22.4 | 27.7 | 140.1 | 0.4 |
| **3B6** |  | 7.19 | 27 | 0.62 | 180.19 | 31.32 | 45.43 | 5.58 | 59.03 | 40.31 | 1.1 | 5.23 | 0.05 | 3.73 |  |  |  |  |
| 3C6 |  | 7.48 | 96 | 0.31 | 25.17 | 9.18 | 16.87 | 2.4 | 77.02 | 42.64 | 0.62 | 1.27 | 0.05 | - |  |  |  |  |
| 3A12 | d3 12:00 h | 6.68 | 930 | 0.04 | 2141.35 | 23.84 | 338.80 | 37.26 | 3455.22 | 332.51 | 1.35 | 48.19 | 0.05 | - | 44.6 | 11.6 | 116.5 | 1728.3 |
| 3B12 |  | 7.27 | 228 | 0.07 | 473.65 | 77.99 | 69.65 | 10.63 | 769.15 | 121.19 | 0.92 | 18.3 | 0.06 | - |  |  |  |  |
| **3C12** |  | 7.86 | 30 | 0.55 | 18.29 | 6.75 | 14.02 | 3.07 | 33.11 | 17.48 | 0.65 | 1.62 | 0.05 | 3.49 |  |  |  |  |
| 3A18 | d3 18:00 h | 7.08 | 88 | 0.96 | 142.50 | 47.33 | 61.91 | 6.63 | 217.75 | 182.43 | 0.83 | 1.79 | 0.04 | - | 40.1 | 19.0 | 139.7 | 6.5 |
| 3B18 |  | 6.56 | 856 | 0.02 | 2203.3 | 120.04 | 257.95 | 27.33 | 4851.71 | 221.46 | 1.83 | 33.68 | 0.05 | - |  |  |  |  |
| **3C18** |  | 7.69 | 60 | 0.44 | 51.39 | 12.01 | 13.24 | 3.16 | 67.3 | 48.22 | 0.69 | 1.53 | 0.04 | - |  |  |  |  |
| 3A24 | d3 24:00 h | 7.66 | 24 | 0.38 | 25.32 | 10.44 | 15.79 | 2.51 | 34.55 | 43.14 | 0.61 | 0.95 | 0.04 | 3.17 | 27.9 | 19.3 | 145.1 | 0 |
| **3B24** |  | 7.32 | 33 | 0.64 | 37.09 | 10.79 | 28.95 | 3.24 | 77.74 | 38.23 | 0.8 | 1.79 | 0.05 | - |  |  |  |  |
| 3C24 |  | 7.36 | 22 | 0.41 | 19.84 | 6.98 | 21.62 | 2.95 | 34.55 | 45.11 | 0.81 | 1.39 | 0.05 | - |  |  |  |  |

Table S2: Physicochemical and climatic characteristics of sampled sectors (see Figure S1). The IDs of sectors selected for soil RNA extraction are indicated in bold and underlined.

| Sample | Reads | RefSeq hits | KEGG hits |
| --- | --- | --- | --- |
| d1 06:00h | 15,870 | 2001 | 943 |
| d1 12:00h | 19,657,220 | 2,380,513 | 1,195,836 |
| d1 18:00h | 18,285,507 | 2,175,145 | 1,043,011 |
| d1 24:00h | 4,876,860 | 349,654 | 169,798 |
| d2 06:00h | 4,689,189 | 402,681 | 194,628 |
| d2 12:00h | 27,834,164 | 4,494,169 | 2,311,516 |
| d2 18:00h | 38,278,971 | 4,429,521 | 2,169,855 |
| d2 24:00h | 85,992,016 | 11,869,036 | 5,209,817 |
| d3 06:00h | 92,971 | 11,222 | 5,384 |
| d3 12:00h | 25,419,489 | 2,650,874 | 1,323,297 |
| d3 18:00h | 14,457,719 | 1,381,969 | 702,831 |
| d3 24:00h | 28,715,655 | 3,772,491 | 1,939,953 |
| Total | 268,315,631 | 33,919,276 | 16,266,869 |

Table S3: library sequence output and mapping statistics. RefSeq and KEGG mapped reads were calculated at phylum and pathway levels, respectively

| Ortholog | EC ID | KEGG pathway | logFC | logCPM | F | FDR p-value |
| --- | --- | --- | --- | --- | --- | --- |
| ANP1; mannan polymerase II complex ANP1 subunit | 2.4.1.- | Various types of N-glycan biosynthesis | 7.177 | 6.637 | 29.195 | 2.48E-04 |
| HSP90B. TRA1; heat shock protein 90kDa beta |  | PI3K-Akt signaling pathway | 7.140 | 6.930 | 29.524 | 2.48E-04 |
| K14274; D-xylonolactonase |  | Pentose and glucuronate interconversions | 6.311 | 5.282 | 18.425 | 1.34E-02 |
| pabBC; para-aminobenzoate synthetase / 4-amino-4-deoxychorismate lyase | 2.6.1.85 4.1.3.38 | Folate biosynthesis | 6.045 | 4.821 | 15.695 | 2.76E-02 |
| CMPK1. UMPK; UMP-CMP kinase | 2.7.4.- 2.7.4.14 | Pyrimidine metabolism | 5.103 | 6.725 | 17.544 | 1.70E-02 |
| [hydrolases of carbon-halide compounds] | 3.8.1.- | Chloroalkane and chloroalkene degradation | 5.040 | 5.931 | 15.264 | 2.83E-02 |
| RP-L29e. RPL29; large subunit ribosomal protein L29e |  | Ribosome | 4.820 | 6.230 | 15.045 | 2.83E-02 |
| Homocitrate synthase | 2.3.3.14 | Lysine biosynthesis | 4.706 | 7.798 | 23.250 | 1.78E-03 |
| DNAH; dynein heavy chain. axonemal |  | Fagosome | 4.185 | 7.166 | 15.754 | 2.76E-02 |
| MYH; myosin heavy chain |  | Tight junction | 4.152 | 6.957 | 14.439 | 3.51E-02 |
| recJ; single-stranded-DNA-specific exonuclease | 3.1.-.- | Base excision repair | 3.569 | 7.751 | 15.208 | 2.83E-02 |
| SF3B1. SAP155; splicing factor 3B subunit 1 |  | Spliceosome | 3.355 | 8.229 | 16.329 | 2.61E-02 |
| TUBB; tubulin beta |  | Gap junction | 3.326 | 9.247 | 27.790 | 3.08E-04 |

Table S4: Significantly differentially transcribed KEGG orthologs between grouped day (12:00 and 18:00 h, n=6) and night (6:00 and 24:00 h, n=5) metatranscriptome libraries, sorted by log_2_ fold-change. Statistical significance threshold of FDR-corrected p-values ≤0.05 for a generalized linear model (glm) quasi-likelihood fit computed with EdgeR (*glmQLFit* function). logFC and logCPM indicate log_2_ fold-changes and average log_2_ counts per million, respectively; F shows the result of the quasi-likelihood F-test.
